# An Integrated Machine Learning Algorithm for Separating the Long-Term Deflection Data of Prestressed Concrete Bridges

**DOI:** 10.3390/s18114070

**Published:** 2018-11-21

**Authors:** Xijun Ye, Xueshuai Chen, Yaxiong Lei, Jiangchao Fan, Liu Mei

**Affiliations:** 1 School of Civil Engineering, Guangzhou University, Guangzhou 510006, China; xijun_ye@gzhu.edu.cn (X.Y.); 2111716068@e.gzhu.edu.cn (X.C.); 2111716142@e.gzhu.edu.cn (Y.L.); 2111716102@e.gzhu.edu.cn (J.F.); 2 Guangdong Provincial Key Laboratory of Durability for Marine Civil Engineering, Shenzhen University, Shenzhen 518060, China

**Keywords:** machine learning, deflection signal separation, Butterworth filter, EEMD, PCA, FastICA

## Abstract

Deflection is one of the key indexes for the safety evaluation of bridge structures. In reality, due to the changing operational and environmental conditions, the deflection signals measured by structural health monitoring systems are greatly affected. These ambient changes in the system often cover subtle changes in the vibration signals caused by damage to the system. The deflection signals of prestressed concrete (PC) bridges are regarded as the superposition of different effects, including concrete shrinkage, creep, prestress loss, material deterioration, temperature effects, and live load effects. According to multiscale analysis theory of the long-term deflection signal, in this paper, an integrated machine learning algorithm that combines a Butterworth filter, ensemble empirical mode decomposition (EEMD), principle component analysis (PCA), and fast independent component analysis (FastICA) is proposed for separating the individual deflection components from a measured single channel deflection signal. The proposed algorithm consists of four stages: (1) the live load effect, which is a high-frequency signal, is separated from the raw signal by a Butterworth filter; (2) the EEMD algorithm is used to extract the intrinsic mode function (IMF) components; (3) these IMFs are utilized as input in the PCA model and some uncorrelated and dominant basis components are extracted; and (4) FastICA is applied to derive the independent deflection component. The simulated results show that each individual deflection component can be successfully separated when the noise level is under 10%. Verified by a practical application, the algorithm is feasible for extracting the structural deflection (including concrete shrinkage, creep, and prestress loss) only caused by structural damage or material deterioration.

## 1. Introduction

Due to their advantages of simple calculations, convenient construction and low maintenance costs, long-span prestressed concrete (PC) bridges with 100–300 m spans have been widely designed and built around the world [1]. In recent years, many long-span PC bridges, especially those whose spans are more than 200 m, have suffered from excessive midspan deflection, which can affect their safety during operation and restrict the development of PC bridges with larger spans [2,3,4]. The Koror–Babeldaob Bridge, with a record span of 241 m (791 ft), experienced one of the most famous bridge collapse disasters due to excessive midspan deflection. Built in 1977, this bridge developed a midspan deflection of 1.61 m (5.3 ft) after 18 years’ operation and eventually collapsed in 1996 [5,6]. Deflection, which accurately reflects the structural performance under various loadings and environmental conditions, is one of the key indexes in the safety evaluation of bridge structures. According to multiscale analysis theory [7], the measured deflection data obtained from a bridge health monitoring system can be regarded as the superposition of different effects, such as concrete shrinkage, creep, prestress loss, material deterioration, temperature effects, and live load effects [8,9]. Numerous studies have been conducted on the causes of deflection, deflection prevention measures, and the influencing factors on the deflection of PC bridges. Most of the previous studies used a finite element (FE) method to build an FE model of PC bridges, then the main effects (e.g., concrete shrinkage, creep, prestress loss, structural damage, temperature effect, and live load effect) were exerted on the FE model to calculate the corresponding deflection values [6,10,11,12,13,14]. However, in reality, the deterioration of specific bridges and environmental factors can hardly be accurately simulated. Possible reasons behind this problem include the following [15]: (1) The models of shrinkage and creep of concrete are not accurate; (2) The calculation method of current codes underestimates the long-term prestress loss of prestressed tendons; (3) In existing design approaches, the effects of shear lag and additional curvature due to differential shrinkage and creep between different parts of a box-girder section are neglected when analyzing isolated beam elements. (4) The numerical analysis strategies for deflection prediction of long-span PC bridges over multiple decades are not suitable. Therefore, it is difficult to accurately calculate the deflection value caused by a specific single influencing factor (load effect).

With the development of computer science and signal processing technology, some works on the separation of the measured deflection data based on statistical methods, wavelet analysis, and machine learning have been conducted in recent years. Based on the analysis of long-term monitoring data, it was concluded in [16,17,18] that there is no general linear relationship between deflection and temperature. Statistical methods were then used to discuss the pattern of the temperature effect, but the deflection component of the temperature effect can hardly be accurately extracted from the monitoring data. The relationship between the natural frequency and temperature can be obtained using a statistical method [19,20], but the accuracy of the results is greatly affected by noise. 

According to multiscale analysis theory [7], wavelet analysis has been increasingly applied for deflection separation. To remove the temperature-induced components from the original strain measurement data, Xia et al. [21,22] developed a wavelet-based multicomponent decomposition method. The decorrelation and perfect reconstruction (PR) characteristics of the discrete wavelet transform are utilized for a multiresolution analysis, and the selection criteria of the physical source extraction is embedded in this method. Ye et al. [23] applied a wavelet multiresolution analysis to present the local stress behaviors caused by the highway loading and the temperature effect during construction and operation periods.

Data-driven approaches, such as machine learning, which allow for learning patterns in the sensor data without any knowledge on the physical characteristics of the structure, have been gaining increasing attention [24]. A variety of machine learning algorithms have been proposed in structural health monitoring. Due to the periodic characteristics of deflection from the temperature effect, a separation method based on a singular value decomposition (SVD) was proposed by Liu et al. [25], but the requirement of the periodic length of the separated signal is not clear. Liu et al. [26] developed an adaptive bandwidth filter method based on a particle swarm optimization algorithm and bandwidth filter to separate the temperature effects. Liu et al. [27,28] applied the least squares support vector machine (LS-SVM) method and multiple least squares support vector machine (M-LS-SVM) method to separate the deflection component of the live load effect and the temperature effect. Peng et al. [29] used a time-series analysis combined with an SVM for landslide displacement prediction. By using principal component analysis (PCA), Yan et al. [30] evaluated the safety condition of a three-span steel bridge. Sohn et al. [31] used auto-associative neural networks (AANN) to eliminate the influence of the temperature effect. The machine learning algorithms mentioned above are verified by numerical simulation and measured data. However, the validation results of these algorithms are good for numerical simulation with low noise level. For measured data, the results are greatly affected by noise. Also, the key parameters in some of these algorithms are sensitive to the results, which need to be optimized to get better results.

To characterize the influences of the individual load effect on the long-term deflection data, this paper presents a single-channel blind separation algorithm for separating the deflection component of live load effects, temperature effects, and structural deflection (including concrete shrinkage, creep, and prestress loss). The proposed algorithm is an integrated machine learning algorithm that combines a Butterworth filter, ensemble empirical mode decomposition (EEMD), PCA, and fast independent component analysis (FastICA). It consists of four stages: (1) The live load effect, which is a high-frequency signal, is separated from the raw signal by a Butterworth filter. (2) The EEMD algorithm is used to extract the intrinsic mode function (IMF) components. (3) These IMFs are then utilized as the inputs of the PCA model, and some uncorrelated and dominant basis components are extracted. (4) Finally, FastICA is applied to derive the independent component, that is, the deflection caused by the individual load effect. For this proposed algorithm, we only need to define the standard deviation and the ensemble number for EEMD and component scores for PCA. The standard deviation and the ensemble number are usually defined as 0.1–0.4 and 50–200, respectively. The general requirements of the component score are set as 95%. These three parameters are not sensitive to the results.

## 2. Multiscale Characteristics of Long-Term Deflection Data

Deflection caused by prestress loss, shrinkage, and creep of concrete is an irreversible trend and will change only after a few months. However, the deflection caused by live loads will change several times in one second. Therefore, the recurrence periods of deflection caused by different effects vary. Due to the multiscale characteristics of different effects, the deflection component of an individual load effect can be determined. As mentioned before, the long-term deflection data (*D*) obtained from the bridge health monitoring system can be regarded as the superposition of concrete shrinkage, creep, prestress loss, material deterioration, temperature effects, and live load effects:

(1) $$D=D_{L}+D_{T}+D_{C}+D_{s}+D_{P}+D_{D}+D_{n}$$

where $D_{L}$, $D_{T}$, $D_{C}$, $D_{s}$, $D_{P}$, $D_{D}$, and $D_{n}$ are the deflections caused by live loads, temperature, creep, shrinkage, prestress loss, dead loads, and noise, respectively. Furthermore, $D_{C}$, $D_{s}$, $D_{P}$, and $D_{D}$ compose the structural deflection *D_v_*_,_ which is an irreversible index that can be used to evaluate the condition of a bridge. *D_v_* can be expressed as: align symbols or replace with text versions

(2) $$D_{v}=D_{C}+D_{s}+D_{P}+D_{D}$$

As shown in Table 1, for live loads with deflection ($D_{L}$), there are hundreds of cycles in one second, which is a high frequency. The temperature deflection ($D_{T}$) consists of a daily temperature effect ($D_{T1}$) and an annual temperature effect ($D_{T2}$). Their recurrence periods are 1 day (24 h) and 1 year (8760 h), respectively. They have a medium and low frequency, respectively. For structural deflection (*D_v_*), which will increase each year, the frequency can be considered 0. The amplitude of the live load deflection and temperature deflection is significant in comparison with the yearly increment caused by irreversible structural deflection *D_v_*.

Consequently, the long-term deflection *D* is written as:

(3) $$D=D_{L}+D_{T}+D_{V}+D_{n}$$

Since *D* represents composite data, in order to accurately evaluate the condition of a bridge, an evaluation system for the individual deflection component must be established. Therefore, the separation of the long-term deflection is the prerequisite.

## 3. An Integrated Machine Learning Algorithm

**Figure 1:**
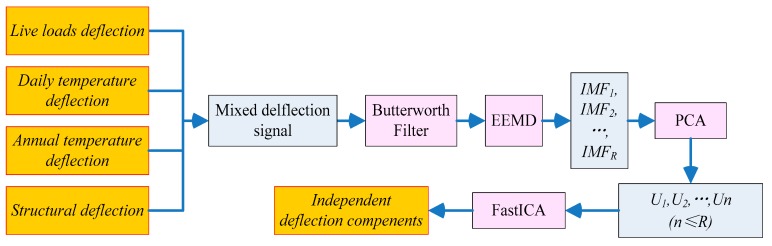
Flowchart of the proposed algorithm.

Figure 1 shows the flowchart and algorithms used in the proposed method. Filtering technology has been widely used in the separation of signals with large time-scale differences, demonstrating a good effect. For the proposed algorithm, in the first step, a Butterworth filter [32] is used to separate the deflection of the live load effect from the long-term deflection. As the daily temperature deflection, annual temperature deflection, and structural deflection belong to a low-frequency domain, there will be a mode mixing problem, so it is difficult to separate the three components by filtering technology.

In the second step, EEMD is used to decompose an observed mixed signal into a collection of IMFs. Although the IMF is a complete, adaptive, and basically orthogonal representation of a signal, EEMD has the characteristics of a binary filter bank structure, similar to dyadic discrete wavelet decomposition. Therefore, the bandwidths of the first few decomposed IMFs are too large, and the IMFs are not completely suitable for using the Hilbert transform to directly obtain an accurate instantaneous frequency spectrum [33,34]. In the third step, these IMFs are utilized as inputs in the PCA model, and some uncorrelated and dominant basis components (*U_i_*) are extracted. The *U_i_* extracted by the PCA algorithm are uncorrelated, but they are not statistically independent. In the last step, a further procedure called FastICA needs to be performed to derive the independent deflection components.

### 3.1. EEMD

As an adaptive signal processing algorithm for nonlinear and nonstationary signals, EMD expresses the signal as the sum of a series of signal components with amplitude-modulated and frequency-modulated parameters, which can reveal the overlap of time and frequency components. To solve the mode mixing problem, a noise-aided data analysis method called EEMD [35] is proposed, which is an extension of the empirical mode decomposition (EMD) [36]. The decomposition principle of EEMD is that, when the signal is added with normally distributed white noise, the signals of different scales are automatically mapped to appropriate scales related to white noise. Different series of normal distributions of Gaussian white noise are added to the signal in several trials. In each trial, added white noise that is different from the decomposed IMFs will show no correlation with the corresponding IMFs from one test to another. If the number of trials (ensemble number) is sufficient, the added noise can be eliminated by averaging all the IMFs produced in each trial [37]. The decomposition process of EEMD is as follows.

(1) Add a white-noise series to the targeted signal.
  (4) $$x_{m}(t)=x(t)+n_{m}(t)$$
  where x(t) is the mixed signal to be analyzed. $n_{m}(t)$ is the added white noise, which has a mean of zero and a standard deviation of σ, which is in the range of 0.1 to 0.4. The index *m* is the ensemble number with a preset maximum number (*m_e_*), which starts from 1.
(2) Decompose the signal $x_{m}(t)$ into a series of IMFs, $c_{i,m},(i=1,2\cdots N-1)$, and a residual, $r_{m}$, using EMD. We can obtain:
  (5) $$x_{1}(t)=\sum_{i=1}^{N-1}c_{i,1}(t)+r_{1}(t)$$
  where *N* − 1 is the total number of IMFs produced in each EMD decomposition.
(3) Repeat Step (1) and Step (2) for *m_e_* trials.
  (6) $$x_{m_{e}}(t)=\sum_{i=1}^{N-1}c_{i,m_{e}}(t)+r_{m_{e}}(t)$$
(4) The final IMFs are obtained by overall averaging the IMFs produced in each trial.
  (7) $$\bar{c_{i}}(t)=\frac{1}{N}\sum_{m=1}^{m_{e}}c_{i,m}(t)$$
  (8) $$\bar{r}(t)=\frac{1}{N}\sum_{m=1}^{m_{e}}r_{m}(t)$$

Therefore, a signal x(t) with multiple components is represented as the sum of some IMFs and a residue:

(9) $$x(t)=\sum_{i=1}^{N-1}{\bar{c}}_{i}(t)+\bar{r}(t)$$

### 3.2. PCA

After the signal is decomposed with the EEMD, *R* IMF components can be obtained. An observation matrix $Y_{R\times L}$ is formed as:

(10) $$Y_{R\times L}={\left[IMF_{1}\;IMF_{1}\;\cdots IMF_{R}\right]}^{T}$$

where *R* is number of IMFs, *L* is length of data, and *T* is the transpose operator.

The observation matrix $Y_{R\times L}$ is then used as the input of the PCA model to find uncorrelated dominant basis components. PCA was developed by Pearson in 1901 [38] as an analogue of the principal axis theorem in mechanics. It was later independently developed and named by Hotelling in the 1930s [39]. In statistics, PCA is a linear transformation method for feature extraction and dimensionality reduction. The principle of PCA is to transform the original correlated random vectors into new noncorrelated random vectors by means of an orthogonal transformation. In geometry, the transform procedure can be expressed as the original coordinate system being transformed into a new orthogonal coordinate system, which points in *P* orthogonal directions where the sample points scatter most openly. Then, a small number of eigenvectors are extracted from it so that these eigenvectors can generalize the most original data information.

In algebra, the transform procedure can be expressed as the covariance matrix of the original random vector being transformed into a diagonal matrix. PCA can be performed by a SVD, usually after a normalization step of the initial data. The SVD of *Y_L×R_* is a factorization of the form $Y_{L\times R}=\left[U_{L\times L}\sum_{L\times R}\;V_{R\times R}^{T}\right]$*,* where *U* and *V* are orthogonal matrices (with orthogonal columns) and ∑ is a matrix of *r* singular values $\lambda_{r}=\lambda_{r\times r}$, where $\lambda_{1}\geq \lambda_{2}\geq \cdots \geq \lambda_{r}\geq 0$. The contribution of each vector is ranked based on the magnitude of its corresponding singular value. The result of the PCA is usually presented by the component score $\beta_{i}$ for each IMF:

(11) $$\beta_{i}=\lambda_{i}/\sum_{i=1}^{n}\lambda_{i}$$

The component score $\sum_{i=1}^{m}\beta_{i}$ refers to the proportion of the variance that each eigenvector represents and can be calculated by dividing the singular value corresponding to that eigenvector by the sum of all singular values. Usually, a component score is required to reach 85% so that the vectors can reflect most of the key information of the original data. More details of PCA can be found in References [40,41].

### 3.3. FastICA

A set of *n* (*n* ≤ *R*) uncorrelated basis IMFs are obtained after applying PCA. The components *U_i_* extracted by the PCA algorithm are uncorrelated, but they are not statistically independent. A further procedure, i.e., ICA, is needed to derive the independent deflection components. ICA [42] is a blind source separation technique that extracts statistically independent components from a set of recorded signals. 

Suppose that *n* independent source signals are received by *m* sensors and that the source signals are randomly mixed through an unknown mixing system to form observation signals. The ICA model can be expressed as:

(12) X(t)=AS(t)

where $X={\left(x_{1},x_{2},\cdots ,x_{m}\right)}^{T}$ represents the observed mixed signal vectors, $S={\left(s_{1},s_{2},\cdots ,s_{n}\right)}^{T}$ is the unknown source signal vectors, and $A={\left(a_{ij}\right)}_{m\times n}$ is an *m× n* dimensional mixed matrix (*m* ≤ *n*). 

**Figure 2:**
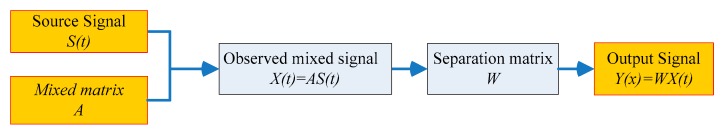
ICA model.

As the source signal S(t) and mixed matrix A are unknown, the goal of ICA is to find a separation matrix *W* so that the output signal Y(t) can be obtained. Y(t) is the best estimation of source signal S(t), and the components of the output signal Y(t) are independent of one another. The ICA algorithm is shown in Figure 2.

The ICA algorithm used in this study is FastICA. The FastICA algorithm (also known as fixed-point ICA) is widely used in signal processing because of its fast convergence speed and good separation effect. The algorithm can estimate the source signals that are independent of one another and mixed by unknown factors from the observed signals. Unlike an ordinary neural network algorithm, FastICA uses batch processing. This means that a large number of sample data participate in each iteration and can be used by applying the fourth-order cumulants, which are the maximum likelihood-based and the maximum entropy-based forms. In addition, this algorithm adopts a fixed-point iterative optimization algorithm, which makes convergence faster and more robust [43,44].

## 4. Numerical Simulation

**Figure 3:**
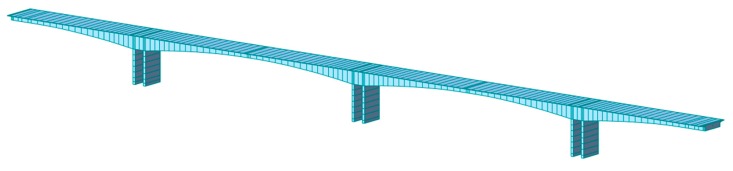
Hanxi Bridge: finite element model in Midas/Civil.

The Hanxi Bridge is a 100 m× 160 m× 100 m prestressed concrete box-girder bridge. The bridge was opened to traffic in 2012 and has been in operation for more than five years. To monitor the safety of the bridge structure, a structural health monitoring system—namely, a liquid level sensing system—was deployed in this bridge to monitor the variations of the girder deflection and ambient temperature in real time. More information on the Hanxi Bridge can be found in Reference [45]. Based on the finite element model developed in Midas/Civil, as shown in Figure 3, the deflections caused by different effects are analyzed.

### 4.1. Characteristic of the Individual Deflection Component of Different Effects

#### 4.1.1. Live Load Effect

**Figure 4:**
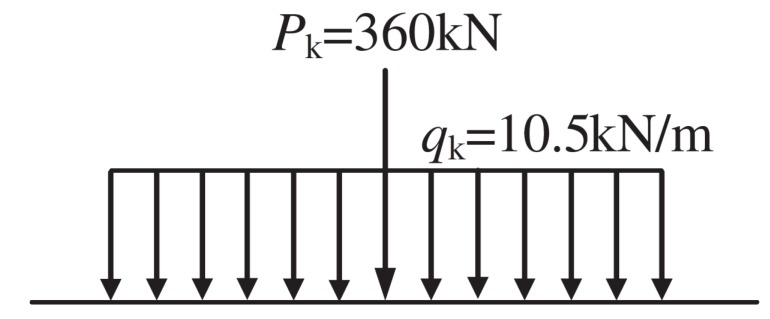
The grade-I lane load of the highway.

The grade-I lane load of a highway is the designed vehicle load of this bridge. As shown in Figure 4, according to General Specifications for Design of Highway Bridges and Culverts (GSDHBC) [46], the grade-I lane load of a highway (which is used for the overall calculation), consists of a concentrated force $P_{k}$ and a distributed force $q_{k}$. However, in order to ensure the safety of the bridge, the grade-I lane load is designed and simplified as the standard vehicle load according to the static strength design (i.e., the dynamic effect is simplified to the static effect) by the influence line loading method. The grade-I lane load is not the actual vehicle load model of the bridge in operation. 

**Figure 5:**
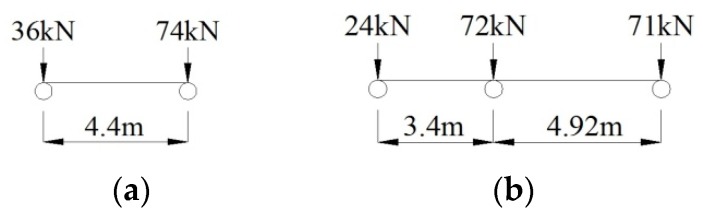
Simplified fatigue-load vehicle model. (**a**) Model *M*_1_*.* (**b**) Model *M*_2_*.*

Based on the survey of the traffic conditions in Guangzhou City, the fatigue-loaded vehicle model was set up based on the simplified load frequency value spectrum by Wang et al. [47]. The simplified fatigue-load vehicle model, which might cause structural damage, consists of two types of vehicles, as shown in Figure 5. Model *M*_1_ is a two-axle vehicle model with a distance of 4.4 m and a total weight of 110 kN, accounting for 16.34% of the total traffic volume. Model *M*_2_ is a three-axle vehicle model with a wheelbase of 3.4 and 4.92 m and a total vehicle weight of 167 kN, accounting for 0.03% of the total traffic volume. Therefore, Model *M_1_* is used to calculate the deflection of the live loads in Midas/Civil software. 

**Figure 6:**
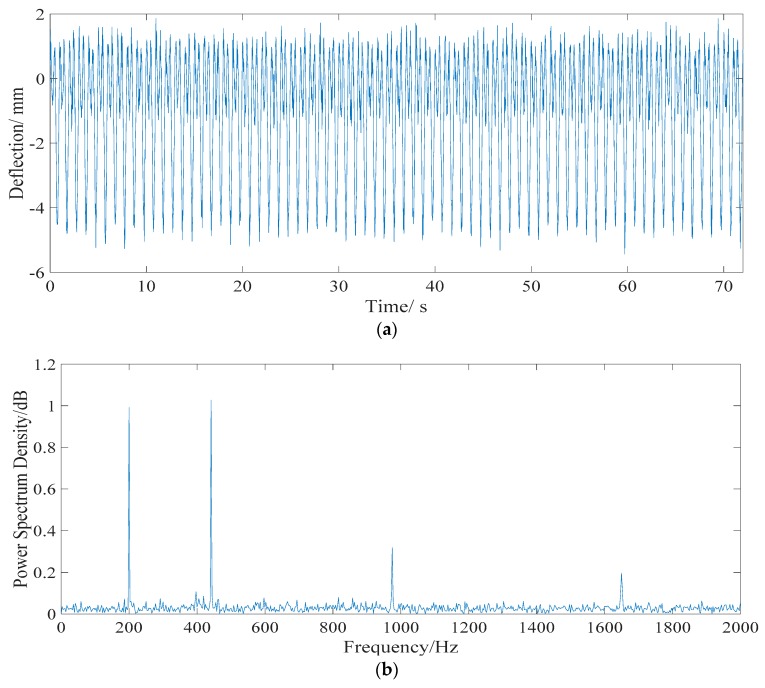
Time history and frequency spectrum of live load deflection. (**a**) Time history data. (**b**) Frequency spectrum.

The simulated signal of live loads deflection, which is a multi-period signal, is collected at a sampling frequency of 4000 Hz. Figure 6a shows the time history of the vehicle load deflection in the midspan of span 2# when Model *M_1_* passes through the bridge at a speed of 60 km/h. The maximum deflection is −5.3 mm. The result of fast Fourier transformation (FFT) is shown in Figure 6b. It is evident that the main frequencies of live loads deflection are greater than 200 Hz, which belongs to the higher-frequency domain. Therefore, the live load deflection effect can be separated by a Butterworth filter.

#### 4.1.2. Temperature Effect

According to GSDHBC [46], the deflection of the temperature effect ($D_{T}$) consists of a daily temperature effect deflection ($D_{T1}$) and annual temperature effect deflection ($D_{T2}$). $D_{T1}$ consists of a daily global temperature effect deflection ($D_{T1-1}$) and daily gradient temperature effect deflection ($D_{T1-2}$). In Guangdong Province, China, the temperature does not vary greatly between day and night. In this paper, the temperature difference between day and night is set as $\Delta_{d1}=10$ °C. For the calculation of deflections related to the gradient temperature effect, an equivalent linear temperature profile can be assumed [48]. The temperature difference between the top and bottom surfaces of the bridge girders is set as 4 °C. $D_{T2}$ is a uniform temperature effect. When calculating the deflection of a bridge structure caused by an annual temperature effect, the temperature of the bridge closure should be set as the reference point to obtain the effective temperature effect at the highest and lowest temperature. The temperature difference of the annual temperature effect is set at $\Delta_{d2}=40\;{}^{\circ}C\left(-20\;{}^{\circ}C\sim +20\;{}^{\circ}C\right)$.Therefore, the temperature effect ($D_{T}$) can be expressed using Equation (13):

(13) $$\left\{ \begin{array}{l} D_{T}=D_{T1}+D_{T2}\\ D_{T1}=D_{T1-1}+D_{T1-2}=\frac{\Delta_{d1}}{2}d_{1}\sin(\frac{\pi t}{12})+\frac{\Delta_{d1}}{2}d_{2}\sin(\frac{\pi t}{12})\\ D_{T2}=\frac{\Delta_{d2}}{2}d_{1}\sin(\frac{\pi t}{4380}) \end{array}\right.$$

**Figure 7:**
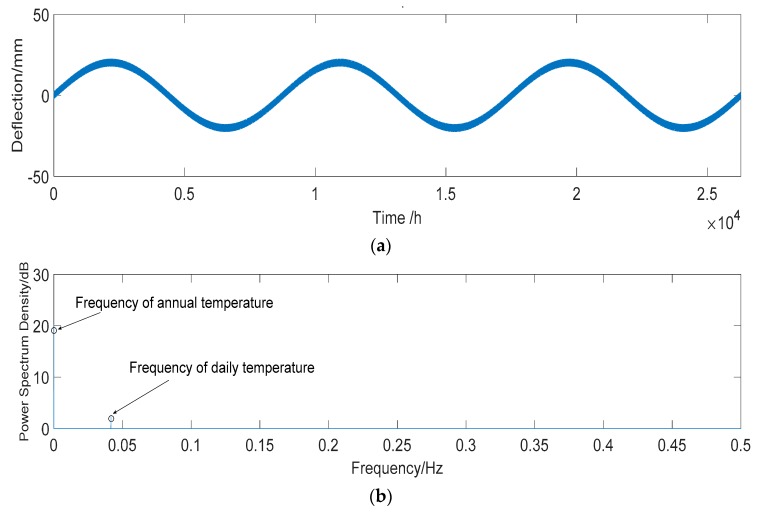
Characteristics of the deflection of temperature effect *D_T_.* (**a**) Three years’ time history of temperature effect deflection *D_T_*. (**b**) Power spectrum of *D_T_*. The sampling frequency is regarded as 1 Hz.

Midas software is used to calculate the deflection of the temperature effect. The results show that the midspan deflection of the main span develops *d*_1_ (+1.48 mm) with a global temperature increase of 1 °C but develops *d*_2_ (−2.92 mm) with an increase in the gradient temperature of 1 °C. It is assumed that there is a linear relationship between the temperature and the deflection of the bridge structure considering the sinusoidal variation. According to Equation (13), the deflection time history of temperature effect deflection can be obtained at a sampling frequency of 1 time/hour, as shown in Figure 7a. The sampling frequency is regarded as 1 Hz. For FFT, the frequency of the daily temperature and annual temperature effects are 0.04 and 0 Hz, respectively.

#### 4.1.3. Effect of Concrete Shrinkage and Creep

**Figure 8:**
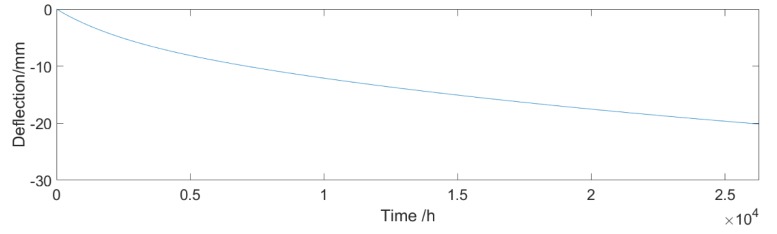
Deflection effect of shrinkage and creep.

The effect of concrete shrinkage and creep, which will be increased by a year, can be considered to have a frequency of 0. In the Code for the Design of Highway Reinforced Concrete and Prestressed Concrete Bridges and Culverts (CDHRCPCBC) [49], the model of CEB-FIP (90) is applied to calculate the effect of concrete shrinkage and creep. The creep coefficient can be expressed as:

(14) $$\left\{ \begin{array}{l} \phi \left(t,t_{0}\right)=\phi_{0}\cdot \beta_{c}\left(t-t_{0}\right)\\ \beta_{c}\left(t-t_{0}\right)=\left[\frac{\left(t-t_{0}\right)/t_{1}}{\beta_{H}+\left(t-t_{0}\right)/t_{1}}\right] \end{array}\right.$$

where $\phi_{0}$ is the nominal creep coefficient, which is related to the loading age of concrete, the relative humidity, and the theoretical thickness of components. $\beta_{H}$ is a coefficient related to the relative humidity and theoretical thickness of components. *t* and *t*_0_ denote the calculating age and loading age of the concrete, respectively. *t*_1_ equals 1 day. The shrinkage strain can be expressed as:

(15) $$\left\{ \begin{array}{l} \varepsilon_{cs}\left(t,t_{s}\right)=\varepsilon_{cs}{}_{0}\cdot \beta_{s}\left(t-t_{s}\right)\\ \beta_{s}\left(t-t_{s}\right)={\left[\frac{\left(t-t_{s}\right)/t_{1}}{350{\left(h/h_{0}\right)}^{2}+\left(t-t_{s}\right)/t_{1}}\right]}^{0.5} \end{array}\right.$$

where $\varepsilon_{cs}{}_{0}$ is the nominal shrinkage coefficient, which is related to the concrete strength and annual mean relative humidity; *h* and *h*_0_ are the theoretical thickness of components and 100 mm, respectively; and *t_s_* is the age of concrete at the beginning of shrinkage. As shown in Figure 8, the bridge develops a deflection of −20.14 mm caused by shrinkage and creep over three years. According to the deflection curve, the expression of fitting curve is obtained by an exponential function, shown as Equation (16):

(16) $$f(t)=D_{c}+D_{s}=-19.72\times e^{\left(0.0001521t\right)}+18.88e^{\left(-0.001833t\right)}$$

#### 4.1.4. Effect of Prestress Loss

**Figure 9:**
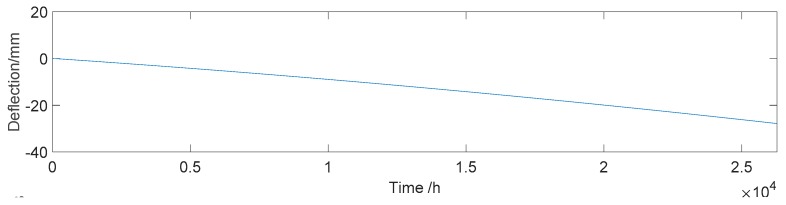
Deflection effect of the prestress loss (*D_p_*).

The results of [50] show that, from the beginning of the operation, a prestress loss of a long-span PC box girder bridge can reach up to 16% in eight years. In this paper, it is assumed that the longitudinal prestress loss of the Hanxi Bridge is 9% in three years, which causes a midspan deflection of −27.8 mm. The deflection caused by prestress loss at any time is obtained by linear interpolation, which is shown in Figure 9.

### 4.2. Validation Result

#### 4.2.1. Total Deflection

As shown in Figure 6, the live load deflection effect signal belongs to the higher-frequency domain and can be separated by a fourth-order Butterworth filter with a 0–2 Hz cutoff frequency. For a signal with a low frequency domain, in order to verify the validity of the proposed algorithm under different noise levels, test noise $D_{n}$ is added to the noise-free signal to obtain the source signal. The sum of temperature effect, shrinkage and creep effect, and prestress loss effect, which is a signal with a low frequency domain, is regarded as the reference noise-free signal to verify the proposed algorithm. In order to verify the validity of the proposed algorithm under different noise levels, test noise *D_n_* is added to the noise-free signal to obtain the source signal. The signal vectors $\hat{s}\left(i\right)$ and x(i) are established as:

(17) $$\left\{ \begin{array}{l} \hat{s}(i)=D_{T}+D_{C}+D_{S}+D_{P}\\ x(i)=\hat{s}(i)+D_{n} \end{array}\right..$$

The signal-to-noise ratio (SNR) can also be estimated as:

(18) $$SNR=10*\mathrm{lg}\left[\frac{P_{S}}{P_{N}}\right]=10*\mathrm{lg}\left[\frac{\sum_{i}^{N}\hat{s}{\left(i\right)}^{2}}{\sum_{i}^{N}\left(x(i)-\hat{s}(i)\right)}\right]$$

where *P_S_* and *P_N_* represent the effective power of the noise-free signal and noise, respectively. x(i) and $\hat{s}(i)$ represent the signal vectors of the source signal data and the reference noise-free signal data, respectively.

#### 4.2.2. Procedure of Separation

**Figure 10:**
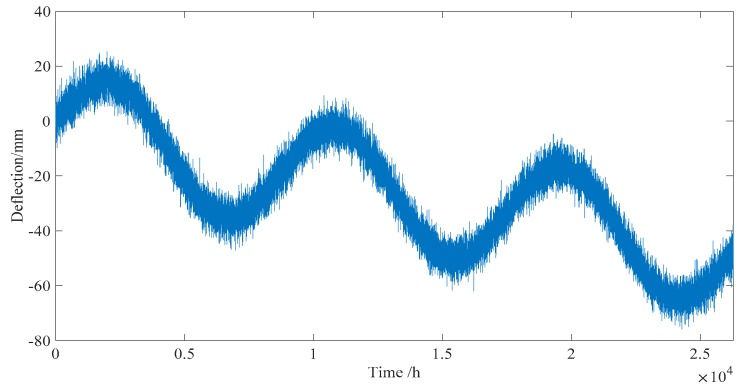
Total deflection of different effects with 10% noise level (not including live load effect).

**Figure 11:**
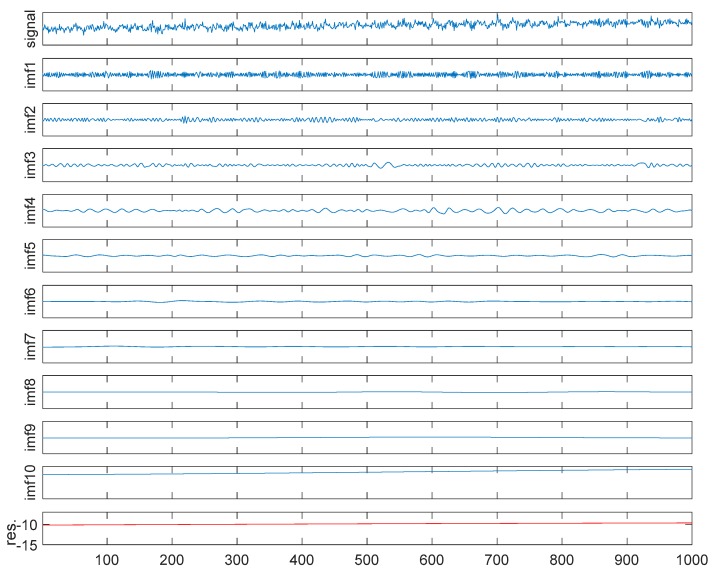
EEMD process.

Due to space limitations, only the signal separation process with a 10% noise level is given here, as shown in Figure 10. At the first stage, we apply the EEMD algorithm on the source single channel signal. Two critical parameters need to be prescribed before the EEMD is applied to a signal. In this case, the standard deviation (σ) and the ensemble number (*m_e_*) are defined as 0.4 and 100, respectively. As a result, we obtained 10 IMFs and a residue (as shown in Figure 11). 

**Figure 12:**
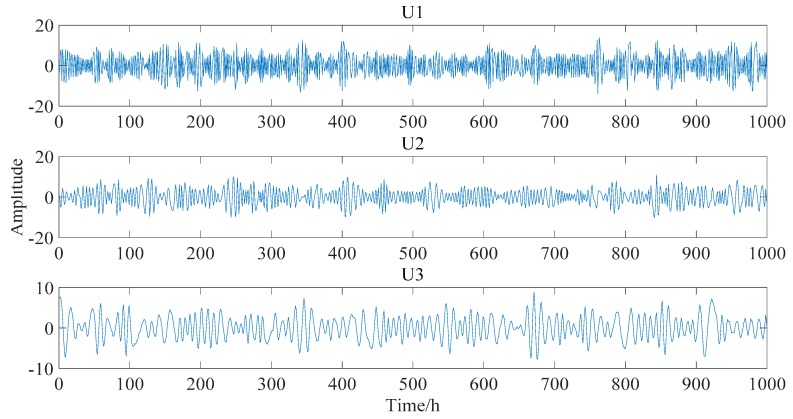
Three basis vectors after PCA.( U1, U2 and U3 are the first three eigenvectors)

**Figure 13:**
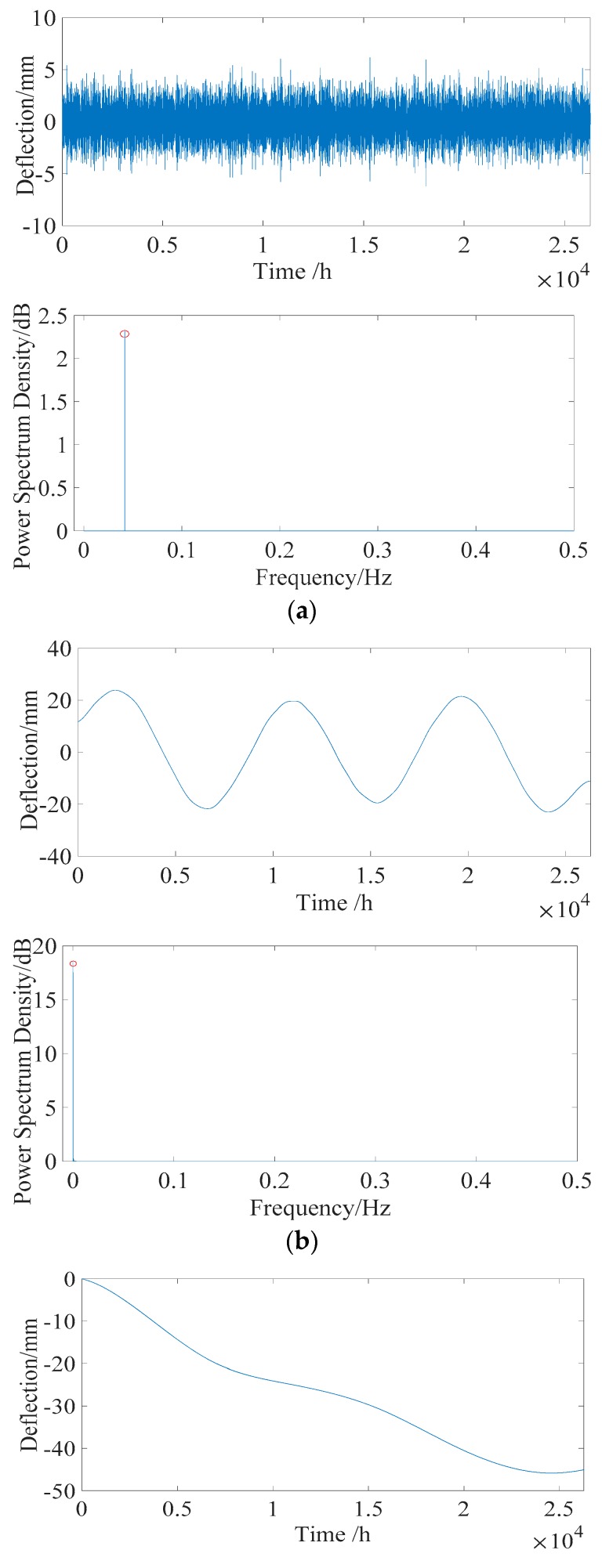

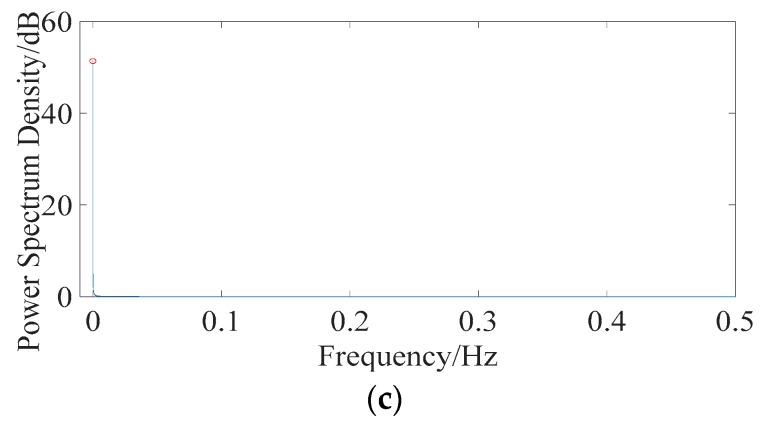
Individual deflection component after separation. (**a**) Daily temperature deflection effect (*D_T_*_1_). (**b**) Annual temperature deflection effect (*D_T_*_2_). (**c**) Structural deflection *D_V_* (including *D_S_, D_C_,* and *D_P_*).

After data normalization, the PCA is applied to the IMF components to obtain uncorrelated basis components. In determining the *r* principal component, the general requirements of the component score are set as 95% in this simulated example. The first three basis vectors, whose component scores are more than 95%, are shown in Figure 12 and are selected as the input of the FastICA model. The estimated output independent deflection components are shown in Figure 13.

To quantitatively analyze the validity of the proposed algorithm, the correlation coefficient ψ is used as an evaluation index to measure the similarity between source signals and the corresponding estimated output components:

(19) $$\psi_{i}=\frac{{\mathrm{cov}(s}_{i}{,z}_{i})}{\sqrt{{\mathrm{cov}(s}_{i}{,s}_{i}{)\mathrm{cov}(z}_{i}{,z}_{i})}}$$

where *s_i_* is the *i-*th source signal component, and *z_i_* is the *i-*th estimated output signal component, shown in Figure 13. The range of the correlation coefficient is [−1, 1]. The closer |ψ| is to 1, the closer the correlation is. Usually, when |ψ| is greater than 0.8, the two vectors are considered to have a high linear correlation. 

The results are shown in Table 2. The signal-to-noise ratio (SNR) is critical to separating the results of the proposed algorithm. For the simulated mixed signals of low frequency domain, the correlation coefficients are greater than 0.84 when the noise level is under 10%. However, when the noise level is greater than 15%, the correlation coefficients are smaller than 0.75. The results show that the proposed algorithm is feasible when the noise level is under 10%. 

## 5. Practical Verification

### 5.1. Description of the Structural Health Monitoring (SHM) System

**Figure 14:**
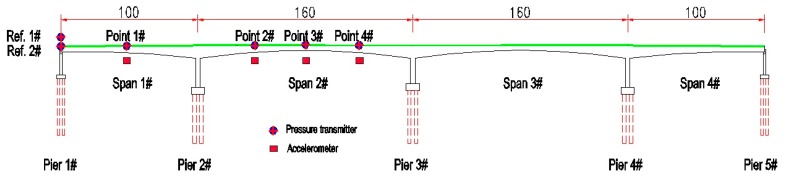
Deployment of the liquid level sensing system (LLSS).

According to the regular inspection report, the Hanxi bridge developed a midspan deflection of −28 mm after four years of operation. As shown in Figure 14, a liquid level sensing system was deployed in 2015. Two reference points were set at pier 1#, and a total of four deflection measurement points were set at span 1# and span 2#. The first three vertical frequencies of the bridge are identified as 0.65, 0.78, and 1.07 Hz from field measurements. The sampling frequency of the liquid level sensing system is set at 4 Hz, which can meet the requirements.

### 5.2. Processing of the Monitored Deflection Data

#### 5.2.1. Live Loads Effect

**Figure 15:**
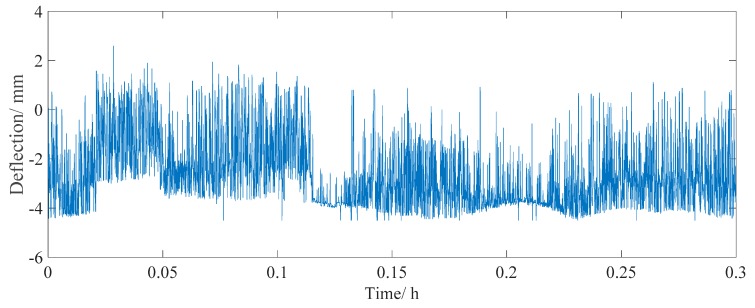
Raw real-time monitored deflection data for half an hour.

The real-time monitoring data of point 3 was used for further analysis. The length of the selected data is one year (from October 2016 to October 2017, lasting for 8760 h, with 126,144,000 total data points). A small data set of the raw signal, which lasts for half an hour, is shown in Figure 15. The maximum/minimum value of the deflection caused by the live load effect was 2 and −4 mm, respectively. Most of the time, the deflection is smaller than −3 mm, which is the deflection caused by light two-axle vehicles traveling on a bridge. When heavier vehicles are driving on the bridge, a greater deflection from −3 to −4 mm is developed. The real-time monitored deflection data can be used as an evaluation index for the overload assessment of vehicles.

**Figure 16:**
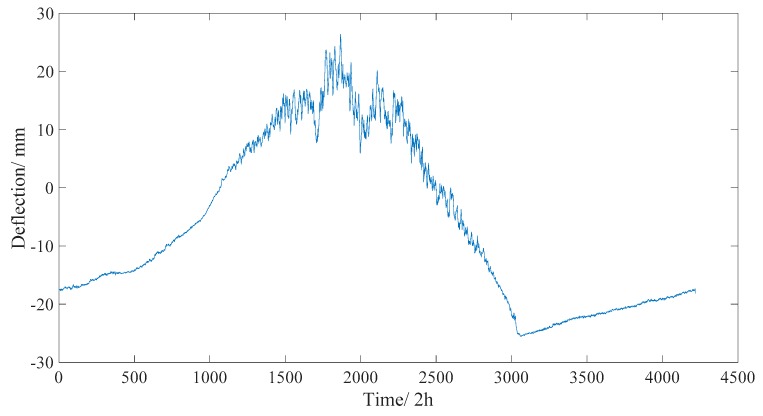
Long-term monitored deflection data of one year (averaged every two hours).

To reduce the storage space on the storage server and save the network bandwidth for data transmission, the real time deflection data of one year is averaged every two hours after separating the live load effect, as shown in Figure 16. The maximum/minimum values of deflection were 25.0 and −23.3 mm, respectively.

#### 5.2.2. Temperature Effect

**Figure 17:**
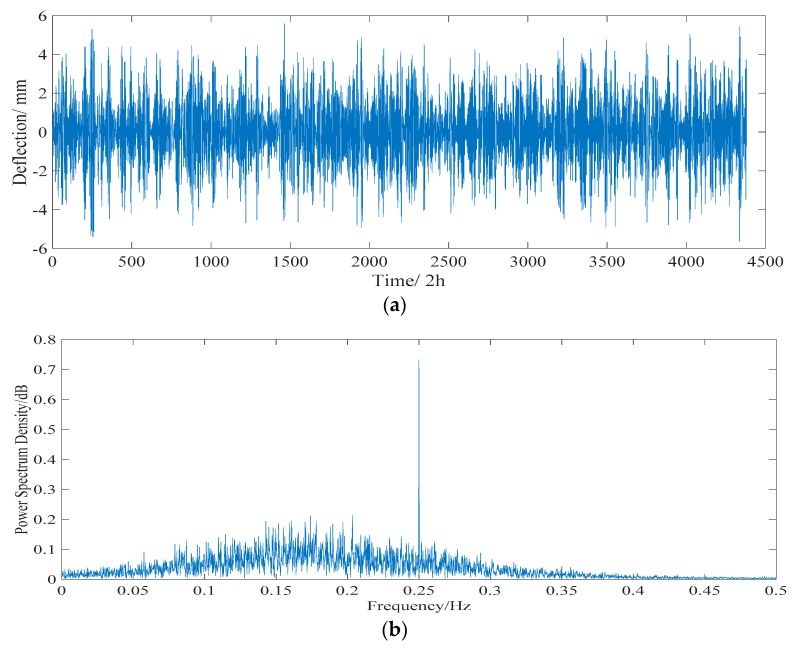
Separated daily temperature deflection effect. (**a**) Time history data. (**b**) Power spectrogram.

**Figure 18:**
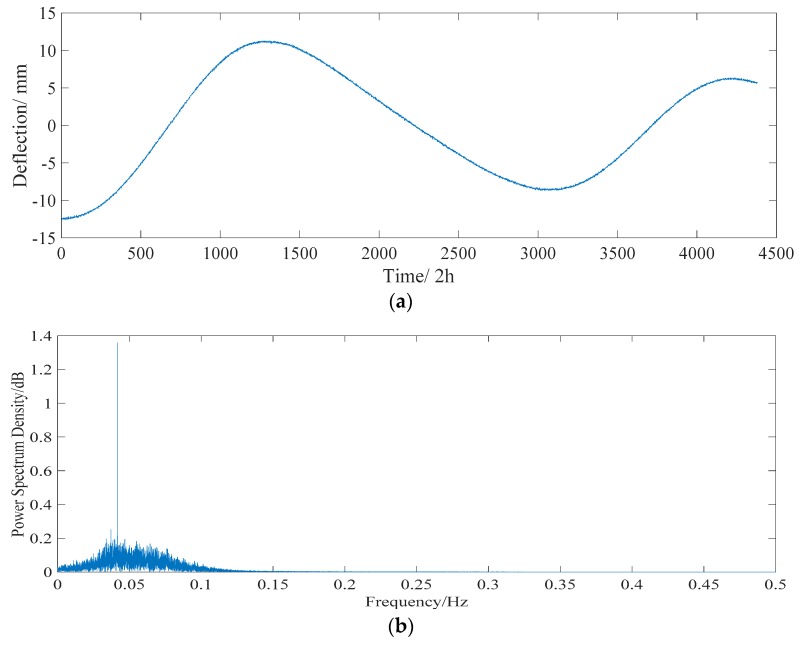
Separated annual temperature deflection effect. (**a**) Time history data. (**b**) Power spectrogram.

According to the proposed algorithm, the long-term monitored deflection data are decomposed into a collection of IMFs by EEMD. These IMFs are utilized as inputs of the PCA model, then some uncorrelated and dominant basis components are extracted. A collection of IMFs, whose component score is more than 95%, is used as input of the ICA model. The separated deflections of the daily temperature effect and annual temperature effect are shown in Figure 17 and Figure 18, respectively. The frequencies of the daily temperature deflection and annual temperature deflection are 0.25 and 0.04 Hz, respectively. With a correlation coefficient of 0.803, the range of the separated daily temperature deflection is −5 to 5 mm. The separated annual temperature deflection (with a correlation coefficient of 0.847) changes from −14 to 14 mm, showing a good correlation with the long-term monitored deflection data (Figure 16).

#### 5.2.3. Structural Deflection

**Figure 19:**
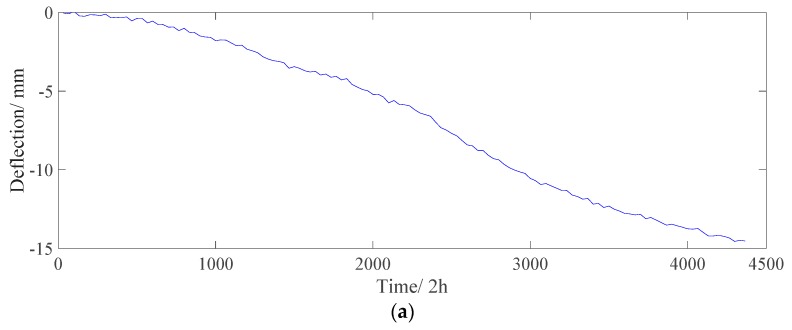

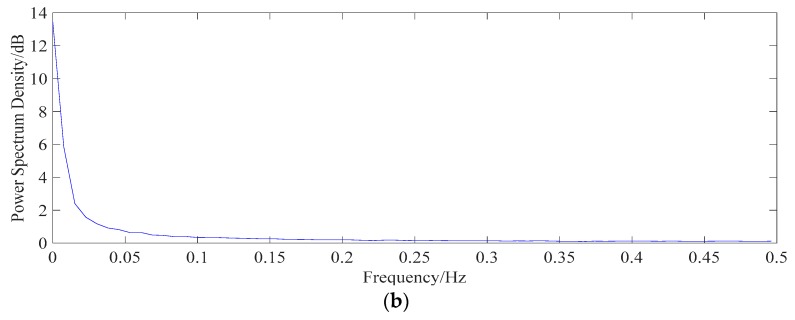
Separated structural deflection effect (including *D_S_, D_C_,* and *D_P_*). (**a**) Time history data. (**b**) Power spectrogram.

For the separated structural deflection, which consists of the effect of creep, shrinkage, prestress loss, and dead load, −14.8 mm developed in one year (from December 2015 to December 2016), as shown in Figure 19. This result indicates that the structural deflection value increases over time and that the bridge might be in a “not healthy” condition. The proposed algorithm can extract the deflection caused only by structural damage and material deterioration.

Thorough regular inspection, several cracks were found in the top slabs, the bottom slabs, and the webs of the box girder, which will directly affect the stiffness of the structure. The interaction between the deflection and cracks makes the deflection much larger than the theoretical value and, with the increase in operating time, the deflection has an increasing trend. Therefore, in order to accurately predict the long-term deflection trend of long-span PC bridges, this interaction should be considered. Furthermore, SHM technology may allow for early warnings but more careful inspections must be conducted to assess the condition of the structure.

## 6. Conclusions

Based on the advantages of EEMD and blind source separation (BSS), an integrated machine learning algorithm consisting of EEMD, PCA, and ICA is presented for estimating the number of sources and separation of the single channel deflection data. The problem of an underdetermined blind source separation is transformed into a well-posed problem, and the deep information contained in the single channel deflection signal is analyzed. By applying the proposed algorithm, different individual deflection components (i.e., live load effect, temperature effect, and structural deflection) can be obtained from the real-time monitoring deflection signal, which can reveal the changing pattern of the structural condition.

The algorithm proposed in this paper is based on the fact that the components of the bridge deflection signal are independent of each other. The separated deflection signal is an ideal mixture of different individual deflection components. The simulated results show that the algorithm is feasible when the noise level is under 10%. However, the algorithm is practicable for field applications under certain conditions. There are some difficulties in processing the real world measured data. For example, in the long-term monitoring data, the sensor output includes not only the deflection signal of the structure, but also the sensor fault caused by the system and environmental conditions (such as bias, drifting, precision degradation, and gain), which will lead to a poor result. Therefore, the detection and correction of the sensor fault is a very important pre-process work for deflection separation. 

It is anticipated that, in the future, more automatic detection and correction algorithms for sensor faults will be proposed and applied to the SHM system. This fact makes the proposed algorithm practical and reliable for the bridge condition assessment of large-span PC bridges in field applications.

## Figures and Tables

**Table 1:** Frequency domain of different effects.

Effects		Frequency Domain		
		Low	Medium	High
Live loads	*D_L_*			
Daily temperature variation (*D_T_*_1_)	*D_T_*			
Annual temperature variation (*D_T_*_2_)				
creep	*D_v_*			
shrinkage				
material deterioration				
noise	-			

Notes: Red, blue and purple color indicate frequency domain of high, medium and low, respectively. Yellow color indicates that noise is distributed in all frequency domains.

**Table 2:** Correlation coefficients (ψ ) between the source signals and estimated components under different noise levels.

Noise Level *(SNR)*	Daily Temperature Effect *D_T_*_1_	Annual Temperature Deflection Effect *D_T_*_2_	Structural Deflection *D_V_*
5%	0.921	0.937	0.916
10%	0.822	0.837	0.804
15%	0.691	0.728	0.703
20%	0.413	0.506	0.398
